# Influence of vaginal lactoferrin administration on amniotic fluid cytokines and its role against inflammatory complications of pregnancy

**DOI:** 10.1186/s12950-017-0152-9

**Published:** 2017-02-15

**Authors:** Martina Maritati, Manola Comar, Nunzia Zanotta, Silva Seraceni, Alessandro Trentini, Fabrizio Corazza, Fortunato Vesce, Carlo Contini

**Affiliations:** 1 Section of Infectious Diseases, Department of Medical Sciences, University of Ferrara, Ferrara, Italy; 2 Institute for Maternal and Child Health – IRCCS “Burlo Garofolo”, Trieste, Italy; 3 Department of Medical Sciences, University of Trieste, Trieste, Italy; 4 Obstetrics and Gynaecology Unit Hospital of Cento, Ferrara, Italy; 5 Section of Medical Biochemistry, Molecular Biology and Genetics, Department of Biomedical and Specialist Surgical Sciences, University of Ferrara, Ferrara, Italy; 6 Section of Obstetrics and Gynaecology, Department of Morphology, Surgery and Experimental Medicine, University of Ferrara, I 44 100 Ferrara, Italy

**Keywords:** Amniotic fluid, Lactoferrin, Pregnancy, Mediators, Cytokines, Inflammatory complications, Abortion, Amniocentesis

## Abstract

**Background:**

An altered amniotic cytokine profile has been reported in inflammatory pregnancy complications with a leading role for IL-6, a marker of the foetal systemic inflammatory response. Up to this date there is no exhaustive information neither on the foetal cytokine balance nor on the best method for its modulation. We aimed to evaluate the influence of vaginal lactoferrin administration on amniotic fluid concentration of 47 cytokines, chemokines and growth factors.

**Methods:**

Sixty women undergoing genetic amniocentesis were enrolled in an open-label clinical trial. 300 mg of vaginal lactoferrin (Florence, Italy) were randomly administered to obtain 3 groups: A, 20 untreated patients; B and C (20 patients in each) respectively treated 4 and 12 h before amniocentesis. Cytokines, chemokines and growth factors concentrations were quantified by a magnetic bead Luminex multiplex immunoassays panel technology. Data analysis was performed with the software Stata (v. 13.1) and GraphPad Prism (v. 5). Group comparisons were performed using Kruskal–Wallis followed by Mann–Whitney U tests, with Bonferroni correction for multiple comparisons. A *p* < 0.05 was considered significant.

**Results:**

Among the 47 tested mediators, 24 (51.06%) were influenced by lactoferrin. 11 (23.4%), showed a highly significant difference (p <0.001); among these IL-9, IL-15, IFN-γ, IP-10, TNF-α, IL-1α and MCP-3 underwent a down-regulation, while IL-17 and FGF-basic, G-CSF, GM-CSF an up-regulation. Difference between group C and both B and A was small for IL-15, IP-10, IL-1α, MCP-3, while it was negligible for IL-9, IFN-γ and TNF-α. IL-17 and the 3 growth factors were strongly enhanced in B and C groups. IL-17, FGF-basic and GM-CSF showed increasing concentrations in both B and C groups, while G-CSF resulted up-regulated only in group C. Significance was intermediate (*p* < 0.01) for the down regulated IL-2RA, IL-12p40 and IFNα2 (6.38%) while it was small for 10 mediators (21.27%) 7 of which (IL-2, IL-4, eotaxin, PDGF-BB, RANTES, IL-18 and MIF) down-regulated and 3 (MCP-1, IL-3, and SDF-1α) up-regulated.

**Conclusion:**

Lactoferrin down-regulates 17 pro-inflammatory amniotic mediators while up-regulating 7 anti-inflammatory amniotic mediators, 5 of which definitively belonging to an anti-inflammatory profile. These findings open to clinical investigation on its use against inflammatory complications of pregnancy.

## Background

Successful pregnancy depends on an orderly production of trophoblast mediators aimed at modulating maternal adaptation, progressively increasing uterine-placental perfusion to draw substances for foetal growth. This task must be directly performed by the foetus, counteracting the natural reaction against the trophoblast induced uterine blood vessel rupture, that in any other tissue but uterus should activate inflammatory changes leading to coagulation and smooth muscle contraction. Such a foetal capacity must be even stronger in the presence of maternal pathologic conditions linked with inflammation, like rheumatic disease, coagulation, and thrombophilia. With regard to maternal immunity and related mediators, it has been reported that Th1-type is incompatible with successful pregnancy, while Th2-type is protective [1].

As for the foetal role, there is a large body of data on normal trophoblast function [2] as well as evidence indicating that foetal chromosomal abnormalities are characterized by alteration of some mediators of inflammation and coagulation which may explain the high incidence of early abortion linked with aneuploidy [3, 4]. Such evidence reinforces the concept of a direct foetal control on normal gestational processes.

Furthermore, an altered amniotic cytokine profile has been reported in pregnancy complications such as premature delivery and chorioamnionitis with a leading role for IL-6, a marker of the foetal systemic inflammatory response [5]. In this regard, once recognized that an increased concentration of IL-6 in the foetal compartment cannot be considered a benign condition, attempts were made to reduce it by administering antibiotic [6, 7] as well as by lactoferrin (LF) administration [8]. Up to this date there is no exhaustive information neither on the foetal cytokine balance nor on the best method for its modulation, when clinically justified. Therefore, with the aim to contribute to a better understanding of the foetal mediators of physiological pregnancy, we analysed mid-trimester amniotic fluid (AF) concentration of a 47 cytokines panel, in basal condition as well as upon assumption of LF, an 80 kDa iron binding glycoprotein of the transferrin family, component of the mammalian innate immune system provided with anti-inflammatory and antimicrobial properties [9].

## Methods

We performed an open-label clinical study enrolling 60 pregnant patients undergoing genetic amniocentesis at the 16th gestational week at the Obstetric Unit of Ferrara University from March 2011 to March 2012. The inclusion criteria were: singleton physiological pregnancy and maternal age as the only indication to foetal karyotyping. The exclusion criteria were: assumption of drugs interfering with the immune system, previous miscarriages, pregnancy at risk for maternal or foetal disease. The research was carried out in accordance with the ethical principles of the Declaration of Helsinki. The Local Ethics Committee approved the design of this study and all pregnant women gave their written informed consent. Moreover, a questionnaire was administered to the patients in order to check for any complications (vaginal bleeding, uterine contraction, rupture of the membranes) within 7 days following the procedure.

Our protocol entails using a 22-gauge needle provided with a 24-gauge tip to reduce the diameter of the hole in the amniotic membrane and the consequent risk of AF leakage. Eligible patients were randomly assigned in a 1:1:1 ratio with a random number table to receive a vaginal compound containing 300 mg of LF (Difesan, Progine, Italy) to obtain 3 groups:

- group A: 20 untreated patients;
- group B: 20 patients treated 4 h before amniocentesis;
- group C: 20 patients treated 12 h before amniocentesis.

Based on a previous study, the compound was administered in the pharmaceutical form of a tablet by means of an applicator deeply inserted into the vaginal route.

A total amount of 20 ml AF was withdrawn for karyotype analysis, microbiological culture, α-fetoprotein (18 ml) and cytokines analysis.

Fresh AF specimens (1 ml) were centrifuged at 3000 g, 4 °C for 10 min, aliquoted and stored at −80 ° C until analysis. In supernatant specimens diluted (1:4) and tested in triplicate, the chemokines and cytokines concentrations were quantified by a magnetic bead Luminex multiplex immunoassays panel technology (47 analytes) (Bio-Plex, BIORAD Laboratories, Milan, Italy), as previously described [10]. The samples were treated following manufactures’ instructions, using the Bio-Plex array reader (Luminex, Austin, TX) to determine the cytokines levels expressed as concentration (pg/mL) by the Manager software. The validation of the Bioplex platform has been performed in our laboratory using the sandwich ELISA assay as the reference [11].

Data analysis was performed with the software Stata (v. 13.1) and GraphPad Prism (v. 5). Group comparisons were performed using Kruskal–Wallis followed by Mann–Whitney U tests, with Bonferroni correction for multiple comparisons. A *p* < 0.05 was considered significant.

## Results

The mean maternal age in the whole study population was 37.5 ± 2.1 years (minimum 35, maximum 42). This parameter was not different among the 3 groups (p = 0.5) and distributed as follows: 37.8 (±2.4), 37.7 (±2.3) and 37.1 (±2.1) years, for groups A, B and C, respectively.

**Fig. 1:**
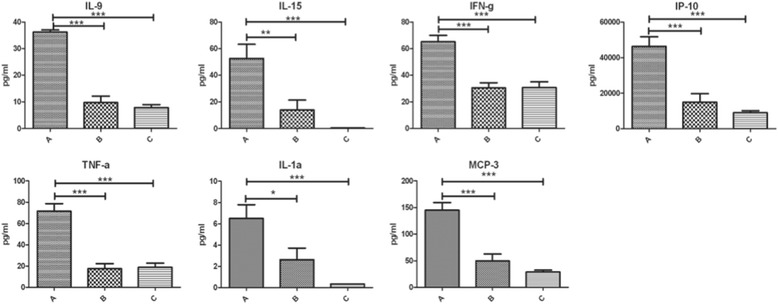
Differences among strongly down-regulated mediators in the three groups. Legend: Data are given as medians (quartiles) (pg/ml); ***p < 0.001; **p < 0.01; *p < 0.05

**Fig. 2:**
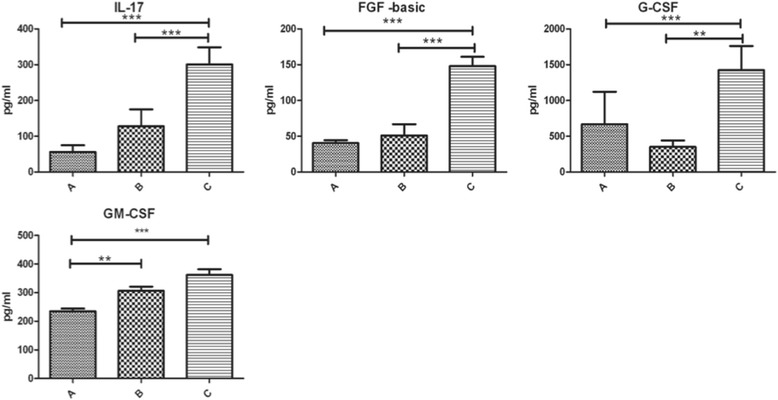
Differences among strongly up-regulated mediators in the three groups. Legend: Data are given as medians (quartiles) (pg/ml); ***p < 0.001; **p < 0.01; *p < 0.05

A normal karyotype was registered in all cases. The AF microbiological cultures were negative in all the tested samples. No complications were registered within 7 days after amniocentesis, and the course of pregnancy was normal in all patients, ending in spontaneous delivery at term. Among the 47 tested mediators, 24 (51.06%) were influenced by LF administration although with different levels of statistically significant difference. In particular, 11 (23.4%), showed a highly significant difference (p <0.001); among these, 7 mediators (IL-9, IL-15, IFN-γ, IP-10, TNF-α, IL-1α, MCP-3) underwent a down-regulation, 4 (IL-17 and 3 factors FGF-basic growth, G-CSF, GM-CSF) an up-regulation. The statistical difference between group C and both B and A was small for IL-15, IP-10, IL-1α, MCP-3, while it was negligible for IL-9, IFN-γ and TNF-α. (Table 1, Fig. 1). As for the strongly up-regulated ones, IL-17 and 3 growth factors (FGF-basic, G-CSF, GM-CSF) were enhanced in groups B and C. In particular IL-17, FGF-basic and GM-CSF showed increasing concentrations in both groups B and C, while G-CSF resulted up-regulated in group C only (Table 1, Fig. 2).

**Table 1:** Amniotic fluid concentration of mediators showing statistically significant difference in the three groups of examined patients

Cytokines		Group A	Group B	Group C
Down-regulated	IL-9***	35.69 (33.96–50.89)^a,d^	5.89 (0.15–14.31)	7.52 (4.61–10.01)
	TNF-α***	61.63 (56.49–72.93)^a,d^	1.63 (1.63–30.97)	19.99 (1.63–29.06)
	IP-10***	38964 (27318–108516)^a,d^	7739 (4945–14536)	8732 (5422–12633)
	IFN-γ***	66.84 (51.9–76.64)^a,d^	24.7 (17.66–44.11)	27.7 (12.88–47.51)
	MCP-3***	174.3 (89.8–216.3)^a,d^	28.63 (22.13–44.32)	30.49 (23.79–41.70)
	IL-15***	81.27 (0.42–99.4) ^a,e^	0.42 (0.42–0.42)	0.42 (0.42–0.42)
	IL-1α***	7.9 (0.35–11.75)^c,d^	0.35 (0.35–0.35)	0.35 (0.35–0.35)
	IL-2RA**	1659 (771,5–2414)^e^	800.9 (426.2–3125)	5957 (496.2–854.5)
	Il-12p40**	1809 (1170–2518)^e^	1.17 (1.17–917)	1.17 (1.17–1.17)
	IFN-α2**	195.1 (128.9–233.1)^c,e^	127.3 (103.2–146.8)	120.1 (98.18–135)
	IL-2*	30.04 (0.2–35.02)^c,f^	0.2 (0.2–2.95)	0.2 (0.2–4.1)
	IL-4*	1.97 (0.06–2.6)^c^	0.06 (0.06–1.475)	1.09 (0.06–2.1)
	EOTAXIN*	300.4 (11.49–432.1)^c,f^	6.01 (0.48–23.2)	15.67 (0.48–31.28)
	PDGF-BB*	89.13 (53.04–122.1)^c^	39.86 (30.24–70.64)	50.43 (30.62–95.22)
	RANTES*	37.75 (7.07–42.58)^c^	0.52 (0.52–32.96)	21.37 (0.52–39.84)
	Il-18*	240 (82.3–336.6)^f^	98.97 (47.37–263.3)	67.72 (52.95–164.1)
	MIF*	2155 (1587–3005)^c^	1440 (1063–1804)	1446 (1098–2232)
Up-regulated	Il-17***	37.02 (32.75–45.11)^d^	0.44 (0.44–205.5) ^g^	302.8 (100–480.6)
	FGF-b***	36.59 (34.57–39.21)^d^	0.25 (0.25–105.8) ^g^	100 (99–202.2)
	G-CSF***	115.5 (45.26–354)^d^	162 (43.92–599.8) ^h^	742.1 (362–1943)
	GM-CSF***	231 (201.8–253.8)^b^	300 (284.2–325.8)	350.5 (300–411.4)
	MCP-1*	167.8 (78.68–386–7)^f^	401.2 (215.6–515.5)	374.6 (258.4–695.2)
	IL-3*	404.7 (321.4–483.7)^c^	537.7 (398.1–697)	537.7 (350.3–612.1)
	SDF-1α*	152 (129.8–661.2)^f^	541 (261.1–703.5)	617 (541.3–711.8)

Data are given as medians (quartiles) (pg/ml); ***p < 0.001; **p < 0.01; *p < 0.05

Group A vs Group B: ^a^ *p* < 0.001; ^b^ *p* < 0.01; ^c^ *p* < 0.05

Group A vs Group C: ^d^ *p* < 0.001; ^e^ *p* < 0.01; ^f^ *p* < 0.05

Group B vs Group C: ^g^ *p* < 0.001; ^h^ *p* < 0.01

Three of the mediators analysed (6.38%) IL-2RA, IL-12p40 and IFNα2 were down-regulated and showed an intermediate statistical significance (*p* < 0.01).

Finally, 10 mediators (21.27%) were influenced with a weakly significance (*p* < 0.05). Among these, 7 (IL-2, IL-4, eotaxin, PDGF-BB, RANTES, IL-18 and MIF) were down-regulated and the remaining 3 (MCP-1, IL-3, and SDF-1α) up-regulated (Table 1).

The analysis by ELISA confirmed the concentration trend observed with the BIO-plex Luminex XMap analysis. Although quantitative differences were found between absolute values for the cytokines obtained by Luminex and ELISA assays are found, the relative values are comparable and the two methods have shown similar trends, as also reported in recent literature [12–14].

## Discussion

Given the key-role of foetal mediators in the regulation of pregnancy, a better knowledge of their normal profile and their possible pharmacologic modulation is needed. As we previously demonstrated that vaginal LF administration significantly down-regulates amniotic IL-6 in women undergoing genetic amniocentesis [8], we found it noteworthy to extend our observation to other cytokines, chemokines and growth factors in basal condition as well as upon the action of the glycoprotein.

By analysing our data, 24 out of 47 (51.06%) tested mediators were significantly influenced by LF. Among the group with the highest statistical significance (*p* < 0.001) comprising of 11 analytes, 7 were down-regulated (IL-9, IL-15, IFN-γ, IP-10, TNF-α, IL-1α, MCP-3) while the remaining 4 (IL-17 FGF-basic, G-CSF, GM-CSF) were up-regulated. Most of the down-regulated ones belong to the pro-inflammatory type. Although IL-9 is more frequently associated with allergy, its role in Th1-mediated inflammation has been recently reported. As for IL-15, it is present in the amniochorion and decidua, and increased amniotic concentration was found in preterm labour [15]. Furthermore, the increased amniotic level of the traditional inflammatory cytokines IL-1a, TNF-α, IFN-γ as well as of IL-1beta, IL-4, IL-6, IL-8 and MCP-1 are believed to represent a marker for identification of the patients at risk of preterm delivery [16]. Together with physiology of gestation, the above mediators also control foetal health. In fact, it has been reported that an increased level of pro-inflammatory cytokines TNF-α, IL-1, IL-6, IL-11, as well as VEGF, TGF-α and TGF-β, may damage the foetal capillary endothelium and alveolar epithelium, resulting in hyaline membrane formation. This evidence demonstrates that the cause of hyaline membrane disease is not prematurity per se, but rather inflammatory conditions leading to both premature delivery and foetal disease.

Accordingly, the inflammatory role of IL-1 has also been experimentally documented on sheep by intra-amniotic injection of the recombinant ovine cytokine, which induces a strong elevation of the intestinal mRNA levels for IFN-γ, TNF-α, IL-4 and IL-10. This increased pro-inflammatory state is associated with a decreased level of the intestinal fatty acid binding protein, disruption of the tight junctional protein ZO-1 and loss of ileum mucosal barrier [17]. Only two chemokines were found to be strongly down-regulated, namely IP-10 and MCP-3.

Of note, the mean levels of IP-10 we measured are almost 5 times higher than in previous reports [18]. The observed discrepancy may be due to the different methodology used, since Luminex assay showed higher absolute cytokine values than ELISA, with a good correlation and a similar trend between the two assays. (12-13-14). Nevertheless, since IP-10 is a pro-inflammatory chemokine, the high value recovered in the control group (A) may be related to a physiological response to asymptomatic infection, not diagnosed by routine microbiological culture. In fact, as shown in our study, IP-10 was found to be strongly down-regulated after LF intake, a glycoprotein with anti-inflammatory and antimicrobial properties.

Increased amniotic fluid level of IP-10 has been reported in presence of foetal Down Syndrome, suggesting a link with the increased abortion rate observed in aneuploid pregnancy [19]. Accordingly, a possible role in adverse events of pregnancy, was suggested for other inflammatory cytokines in foetal aneuploidies [3, 4]. Finally, high levels of TNF-α and MCP-3 have recently been observed in AF of women with preterm birth [20]. Such evidence suggests a possible beneficial role for both chemokines down-regulated by LF.

Among the strongly up-regulated mediators there were 3 growth factors, FGF-basic, G-CSF, GM-CSF and the cytokine IL-17. The latter belongs to a complex family including six more members, namely IL-17/IL-17A, IL-17B, IL-17C, IL-17D, IL-17E/IL-25, produced by several different cell types and primarily prompting Th2- and Th9-type responses. Some of these components activate IL-4, IL-5 and IL-13 expression, as well as eosinophil recruitment. In particular, IL-17 has been linked to inflammatory conditions of pregnancy such as chorioamnionitis leading to preterm delivery [21]. Although our data showed an up-regulation of IL-17 12 h after LF intake when compared to the control group thus suggesting a possible detrimental effect of the treatment, it is noteworthy that at 4 h the difference in the cytokine level was no longer visible. It should be noted that the main effects of LF intake, in terms of statistical significance, are observed at 4 h for most of the tested cytokines, as demonstrated before [8]. Thus, this possible discrepancy between the results observed at 4 h and at 12 h might be attributable to sampling characteristics.

As for the 3 growth factors evaluated in our study, little is known about their behaviour during pregnancy. FGF-basic, has been reported to stimulate the growth of cultured amniotic fluid cells, without harmful effects on chromosomes [22]. GM-CSF is a hematopoietic cytokine provided with neurotrophic and neuroprotective functions for which specific pregnancy-stage and organ modulation have been reported in mice foetuses [23]. Finally, G-CSF has been reported to improve pregnancy rate, reducing recurrent miscarriage in assisted reproduction [24].

With regard to the group with intermediate statistical significance, IL-2RA, IL-12p40 and IFN-α2 were all down-regulated. High levels of the first two are linked with pre-eclampsia [25] recurrent miscarriage [26] and preterm birth of growth retarded neonates [27]. Down-regulation of IFN-α2 may possibly be beneficial considering it belongs to the same IFN-γ family. In particular, IFN type I (IFN-α) promotes the expression of T lymphocyte receptors for IL-12, the primary inducer of TH1 population.

The group which showed the lowest statistical significance (*p* < 0.05), includes 10 mediators, among which 7 down-regulated, namely IL-2, IL-4, IL-18, EOTAXIN, RANTES, MIF and PDGF-BB, and 3 up-regulated, i.e. IL-3, SDF-1, MCP-1.

IL-2 and IL-4 belong to the specific immune response of T lymphocytes to protein antigens. Physiologically, they regulate lymphocyte differentiation and growth and activate macrophages and eosinophils, thus producing local tissue damage such as granulomatous inflammation. As for their role during pregnancy, only the receptor for IL-4 (IL-4r), but not the levels of IL-4 and IL-2 have been reported to increase in pre-eclampsia [28].

IL-18, mainly produced by macrophages, belongs to the innate immunity, activating NK and T cells, as well as stimulating IFN-γ production. Its specific function in pregnancy has not been investigated yet, but considering the above-mentioned effects, its decrease following LF administration may be beneficial.

RANTES and Eotaxin are both produced by Th2, although respectively responsible for inflammation and allergy. Provided with pro-invasive function, they are released in the mucosal secretion, needing to be precisely balanced, as an excessive amount can affect the outcome of pregnancy [29, 30]. It is therefore worthy of note that LF is able to reduce their amniotic concentration. The same appears to be true for the MIF produced by the trophoblast at the implantation site, and reported to be linked to pre-eclampsia [31].

With regard to PDGF-BB, this factor is included among the factors regulating the endometrial stromal cell motility at the implantation site [32]. Down-regulation of this factor following LF administration needs extensive clinical investigation.

Among the slightly up-regulated mediators, we found the last three molecules: IL-3, SDF-1 and MCP-1. The first one is a haematopoiesis promoting factor, whose maternal serum level increases as a function of the trimester [33]; the second compound, SDF-1, is a CXC-chemokine expressed by human trophoblast, enhancing VEGF release and possibly facilitating spiral arteries remodelling [34].

The small increase of MCP-1 is more difficult to classify. As reported above, LF induced a highly significant down-regulation of the same chemokine family member MCP-3 and therefore a similar action on MCP-1 was expected. This may be due to the 2 molecules sharing only 70% of the same structure or the distinct receptor usage and spectrum of action: MCP-3 binds to CCR1, CCR2, and CCR3 receptors [33] while MCP-1 binds only CCR2. Accordingly, MCP-3 only partially overlaps MCP-1 action, for instance, the first is active on eosinophils and dendritic cells, which are not affected by MCP-1. Based on such evidence, so far it has not been possible to hypothesize any clinical implication for the small increase of MCP-1 in comparison with the strong decrease of MCP-3.

The maternal immune system undergoes a significant modulation during pregnancy. Historically, it is believed that such adaptation is needed to induce tolerance towards the foetal antigens derived from the inherited paternal genome. However, it became clear that the so called ‘rejection’ of the product of conception does not derive from an antigen-antibody reaction, but rather, even simpler, from an inflammatory one. Indeed, successful pregnancy appears to be assured throughout a delicate balance of pro- and anti-inflammatory cytokines, chemokines and prostanoids, modulating the maternal response towards foetal invasion, but preserving at the same time the capacity to prevent infections from a multitude of potential pathogens. Although maternal mediators shift towards acceptance has not been completely understood, it is known that an anti-inflammatory profile is essential for successful pregnancy, while an inflammatory one can lead to serious complications, including abortion [1], preterm labour, infection and perinatal death [35].

Modulation of maternal immune system is a task of the foetus itself.

Such a logical conviction derives not only from the knowledge of mediators produced by trophoblast [2], but also from studies indicating that foetal aneuploidy - which is by definition linked with pregnancy loss - is characterized by imbalance of cytokines, peptides and prostanoids known to modulate maternal immune response and vascular changes. Indeed, at this regard, while it is admitted that amniotic fluid cytokines may also be maternal [36], those produced in vitro by cytotrophoblast can only be foetal. They represent the tool by which the foetus modulates maternal response, and their imbalance must be considered a feature of the foetus itself [3, 4, 37, 38].

Conversely, since the maternal reaction can also be influenced by pre-existing conditions, the outcome of pregnancy will ultimately depend on the comparison of the foetal ability with the maternal response.

From an anatomy perspective, it can be accepted that vaginally administered LF is absorbed to enter the maternal circulation and it is then transferred to the foetal compartment through the placental barrier, like any other glycoprotein such as maternal antibodies. Not only does ‘Foetal compartment’ mean amniotic fluid, but also foetal body. Therefore, the modulation of amniotic mediator levels can reflect the action of LF on either the foetus or the foetal adnexa.

Obviously, amniotic inflammatory and/or coagulation mediator imbalance can also derive from the mother, especially in a number of chronic connective tissue diseases and other genetic conditions. However, knowing that the trophoblast and the foetus itself do release their own mediators, in the absence of any maternal disease, their imbalance should be interpreted as a direct foetal responsibility. As for the purpose of our study, given the role of the foetal mediators in the control of maternal response, it is noteworthy that LF is able to modulate their balance within the foetal compartment.

The limits of our study were above all the small sample size which needs to be further increased. Secondly, the design of the study didn’t allow to establish the best timing and dosage of LF able to modulate amniotic cytokines to avoid inflammatory complication of pregnancy.

To this purpose, a longitudinal approach would be useful, although these preliminary data represent a good starting point to achieve further experimental results.

## Conclusions

Overall our data shows that vaginal LF administration down-regulates 17 AF pro-inflammatory cytokines while up-regulating 7 mediators, 5 of which definitively belonging to an anti-inflammatory profile. Such a finding reinforces our earlier assertion on a possible protective role of this glycoprotein opening to a more extensive clinical investigation on its use against inflammatory complications of pregnancy.

## Abbreviations

AF: Amniotic fluid
LF: Lactoferrin

## References

[1] Pazos M, Sperling RS, Moran TM, Kraus TA (2012). The influence of pregnancy on systemic immunity. Immunol Res.

[2] Lunghi L, Ferretti ME, Medici S, Biondi C, Vesce F (2007). Control of human trophoblast function. Reprod Biol Endocrinol.

[3] Vesce F, Scapoli C, Giovannini G, Tralli L, Gotti G, Valerio A (2002). Cytokine imbalance in pregnancies with foetal chromosomal abnormalities. Hum Reprod.

[4] Vesce F, Scapoli C, Giovannini G, Piffanelli A, Geurts-Moespot A, Sweep FC (2001). Plasminogen activator system in serum and amniotic fluid of euploid and aneuploid pregnancies. Obstet Gynecol.

[5] Romero R, Gomez R, Ghezzi F, Yoon BH, Mazor M, Edwin SS (1998). A fetal systemic inflammatory response is followed by the spontaneous onset of preterm parturition. Am J Obstet Gynecol.

[6] Vesce F, Buzzi M, Ferretti ME, Pavan B, Bianciotto A, Jorizzo G (1998). Inhibition of amniotic prostaglandin E release by ampicillin. Am J Obstet Gynecol.

[7] Vesce F, Pavan B, Lunghi L, Giovannini G, Scapoli C, Piffanelli A (2004). Inhibition of amniotic interleukin-6 and prostaglandin E2 release by ampicillin. Obstet Gynecol.

[8] Vesce F, Giugliano E, Bignardi S, Cagnazzo E, Colamussi C, Marci R (2014). Vaginal Lactoferrin Administration before genetic amniocentesis decreases amniotic interleukin-6 levels. Gynecol Obstet Invest.

[9] González-Chávez SA, Arévalo-Gallegos S, Rascón-Cruz Q (2009). Lactoferrin: structure, function and applications. Int J Antimicrob Agents.

[10] Zanotta N, Tornesello NM, Annunziata C, Stellato G, Buonaguro FM, Comar M (2016). Candidate soluble immune mediators in young women with high risk of human Papillomavirus infection: high expression of chemokines promoting angiogenesis and cell proliferation. PLoS One.

[11] Comar M, Zanotta N, Bonotti A, Tognon M, Negro C, Cristaudo A, Bovenzi M (2014). Increased levels of C-C chemokine RANTES in asbestos exposed workers and in malignant mesothelioma patients from an hyperendemic area. PLoS One.

[12] Leng SX, McElhaney JE, Walston JD, Xie D, Ferdarko NS, Kuchel GA (2008). ELISA and multiplex technologies for cytokine measurement in inflammation and aging research. J Gerontol A Biol Sci Med Sci.

[13] Codorean E, Nichita C, Albulescu L, Raducan E, Popescu ID, Lonita AC, Albulescu R (2010). Correlation of XMAP and ELISA cytokine profiles; development and validation for immunotoxicological studies in vitro. Roum Arch Microbiol Immunol.

[14] Tighe P, Negm O, Todd I, Fairclough L (2013). Utility, reliability and reproducibility of immunoassay multiplex kits. Methods.

[15] Fortunato SJ, Menon R, Lombardi SJ (1998). IL-15, a novel cytokine produced by human fetal membranes, is elevated in preterm labor. Am J Reprod Immunol.

[16] La Sala GB, Ardizzoni A, Capodanno F, Manca L, Baschieri MC, Soncini E (2012). Protein microarrays on midtrimester amniotic fluids: a novel approach for the diagnosis of early intrauterine inflammation related to preterm delivery. Int J Immunopathol Pharmacol.

[17] Wolfs TG, Kallapur SG, Polglase GR, Pillow JJ, Nitsos I, Newnham JP (2011). IL-1α mediated chorioamnionitis induces depletion of FoxP3+ cells and ileal inflammation in the ovine fetal gut. PLoS One.

[18] Gervasi MT, Romero R, Bracalante G, Erez O, Dong Z, Hassan SS, Yeo L, Yoon BH, Chaiworaponqsa T (2012). Midtrimester amniotic fluid concentrations of interleukin-6 and interferon-gamma-inducible protein-10: evidence for heterogeneity of intra-amniotic inflammation and associations with spontaneous early (<32 weeks) and late (>32 weeks) preterm delivery. J Perinat Med.

[19] Laudanski P, Zbucka-Kretowska M, Charkiewicz K, Wolczynski S, Wojcik D, Charkiewicz R (2014). Maternal plasma and amniotic fluid chemokines screening in fetal Down syndrome. Mediators Inflamm.

[20] Menon R, Bhat G, Saade GR, Spratt H (2014). Multivariate adaptive regression splines analysis to predict biomarkers of spontaneous preterm birth. Acta Obstet Gynecol Scand.

[21] Ito M, Nakashima A, Hidaka T, Okabe M, Bac ND, Ina S (2010). A role for IL-17 in induction of an inflammation at the fetomaternal interface in preterm labour. J Reprod Immunol.

[22] Chettur L, Christensen E, Philip J (1978). Stimulation of amniotic fluid cells by fibroblast growth factor. Clin Genet.

[23] Matsumoto A, Hatta T, Ono A, Hashimoto R, Otani H (2011). Stage-specific changes in the levels of granulocyte-macrophage colony-stimulating factor and its receptor in the biological fluid and organ of mouse foetuses. Congenit Anom.

[24] Cavalcante MB, Costa Fda S, Barini R, Araujo Júnior E (2015). Granulocyte colony-stimulating factor and reproductive medicine: a review. Iran J Reprod Med.

[25] Medeiros LT, Peraçoli JC, Bannwart-Castro CF, Romão M, Weel IC, Golim MA (2014). Monocytes from pregnant women with pre-eclampsia are polarized to a M1 phenotype. Am J Reprod Immunol.

[26] Giannubilo SR, Landi B, Pozzi V, Sartini D, Cecati M, Stortoni P (2012). The involvement of inflammatory cytokines in the pathogenesis of recurrent miscarriage. Cytokine.

[27] Lindner U, Tutdibi E, Binot S, Monz D, Hilgendorff A, Gortner L (2013). Levels of cytokines in umbilical cord blood in small for gestational age preterm infants. Klin Padiatr.

[28] Taylor BD, Tang G, Ness RB, Olsen J, Hougaard DM, Skogstrand K (2016). Mid-pregnancy circulating immune biomarkers in women with preeclampsia and normotensive controls. Pregnancy Hypertens.

[29] Sharma S, Godbole G, Modi D (2016). Decidual control of trophoblast invasion. Am J Reprod Immunol.

[30] Brou L, Almli LM, Pearce BD, Bhat G, Drobek CO, Fortunato S (2012). Dysregulated biomarkers induce distinct pathways in preterm birth. BJOG.

[31] Rolfo A, Giuffrida D, Nuzzo AM, Pierobon D, Cardaropoli S, Piccoli E (2013). Pro-inflammatory profile of preeclamptic placental mesenchymal stromal cells: new insights into the etiopathogenesis of preeclampsia. PLoS One.

[32] Schwenke M, Knöfler M, Velicky P, Weimar CH, Kruse M, Samalecos A (2013). Control of human endometrial stromal cell motility by PDGF-BB, HB-EGF and trophoblast-secreted factors. PLoS One.

[33] Holtan SG, Chen Y, Kaimal R, Creedon DJ, Enninga EA, Nevala WK (2015). Growth modeling of the maternal cytokine milieu throughout normal pregnancy: macrophage-derived chemokine decreases as inflammation/counterregulation increases. J Immunol Res.

[34] Rzepka R, Dołęgowska B, Rajewska A, Kwiatkowski S (2014). On the significance of new biochemical markers for the diagnosis of premature labour. Mediators Inflamm.

[35] Chow SS, Craig ME, Jones CA, Hall B, Catteau J, Lloyd AR (2008). Differences in amniotic fluid and maternal serum cytokine levels in early midtrimester women without evidence of infection. Cytokine.

[36] Zaretsky MV, Alexander JM, Byrd W, Bawdon RE (2004). Transfer of inflammatory cytokines across the placenta. Obstet Gynecol.

[37] Gessi S, Merighi S, Stefanelli A, Mirandola P, Bonfatti A, Fini S, Sensi A, Marci R, Varani K, Borea PA, Vesce F (2012). Downregulation of A(1) and A(2B) adenosine receptors in human trisomy 21 mesenchymal cells from first-trimester chorionic villi. Biochim Biophys Acta.

[38] Vesce F, Farina A, Jorizzo G, Tarabbia C, Calabrese O, Pelizzola D, Giovannini G, Piffanelli A (1996). Raised level of amniotic endothelin in pregnancies with fetal aneuploidy. Fetal Diagn Ther.

